# Supplementary material for: Fronts and waves of actin polymerization in a bistability-based mechanism of circular dorsal ruffles
Source: Nat Commun. 2017 Jun 19;8:15863. doi: 10.1038/ncomms15863 (PMC5481797; doi:10.1038/ncomms15863)
Supplement: Supplementary Information — Supplementary Figures, Supplementary Notes and Supplementary References [file ncomms15863-s1.pdf]

Supplementary Note 1

Secondary wavefronts of arap1 behind wavefronts of actin. Recently, it has been shown that Arap1 forms a secondary wavefront in the interior of the wavefront of polymerized actin in CDRs [1]. Supplementary Fig. 1 shows a kymograph that was sampled from a time-lapse sequence obtained by Hasegawa et al [33] (see specifically Figure 2*A*, therein). High concentrations of Arap1 are only found in the CDR interior. Note that this also holds for the phase of CDR contraction. The relative positions of the maxima of polymerized actin and Arap1 are therefore different between expanding and contracting CDRs; note that for the expanding wavefront there is a pronounced peak of Arap1 following that of actin, whereas for the reversing wave both peaks co-localize, as indicated by the yellowish colors in Supplementary Fig. 1*B*. We found the same phenomenon in the results of our simulations (compare Supplementary Fig. 1*B* to Figure 4*B* and *C* in the main text).

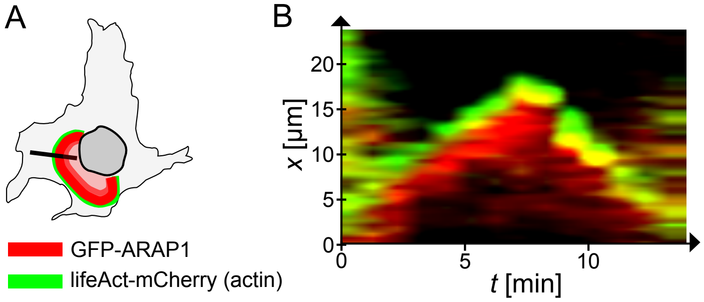

Supplementary Figure 1: *Secondary wavefront of Arap1 in the interior of the wavefront of actin in CDRs.* (*A*) sketch of the cell exhibiting a CDR and the relative positions of the wavefronts of actin (stained by lifeAct-mCherry) and Arap1. The straight black line highlights the position along which the kymograph was (*B*) taken.

Supplementary Note 2

The physical model. The dimensionless Eq. (1)-(4) in the main text have been obtained from a physical model together with a change in variables and parameters.

We consider the following four fields of protein densities

1. branched actin in CDRs (c_b)
2. filamentous actin of stress fibers and the cell cortex (c_f)
3. monomeric actin subunits (c_g)
4. inhibitor of actin polymerization (c_i)

as introduced in the main text (section "bistable actin organization within CDRs") and Figure 2. The model equations read

$$\frac{\partial c_\mathrm{b}}{\partial t} = k_\mathrm{p}\frac{c_\mathrm{b}^2 c_\mathrm{g}}{k_\mathrm{i}+c_\mathrm{i}} - k_\mathrm{d}c_\mathrm{b} + D_\mathrm{b}\Delta c_\mathrm{b} \quad (1a)$$

$$\frac{\partial c_\mathrm{f}}{\partial t} = k_\mathrm{f1}\frac{c_\mathrm{g}}{k_\mathrm{i}+c_\mathrm{i}} - k_\mathrm{f2}c_\mathrm{f} \quad (1b)$$

$$\frac{\partial c_\mathrm{g}}{\partial t} = -k_\mathrm{p}\frac{c_\mathrm{b}^2 c_\mathrm{g}}{k_\mathrm{i}+c_\mathrm{i}} + k_\mathrm{d}c_\mathrm{b} - k_\mathrm{f1}\frac{c_\mathrm{g}}{k_\mathrm{i}+c_\mathrm{i}} + k_\mathrm{f2}c_\mathrm{f} + D_\mathrm{g}\Delta c_\mathrm{g} \quad (1c)$$

$$\frac{\partial c_\mathrm{i}}{\partial t} = k_\mathrm{i1}c_\mathrm{b} - k_\mathrm{i2}c_\mathrm{i} + D_\mathrm{i}\Delta c_\mathrm{i}. \quad (1d)$$

Here k_p describes the kinetics of autocatalytic/cooperative actin polymerization inhibited by the inhibitory complex. The saturation parameter of the latter is given by k_i. Polymerized actin is subject to spontaneous decay and severing, described by the kinetic constant k_d. The diffusivity of branched actin at the membrane (including the corresponding promoting proteins of branching) is given by D_b. The corresponding terms for the spontaneous polymerization of actin of stress fibers and the cell cortex have the kinetic constant of polymerization k_f1 and depolymerization k_f2. The dynamics of the globular actin is conserving the total actin concentration and further considers fast diffusion of actin monomers having a diffusivity of D_g. The inhibitory complex is activated at a rate k_i1 proportional to the concentration of branched CDR actin and subject to spontaneous decay described by the kinetic constant k_i2. The diffusion constant of the inhibitory complex is given by D_i.

To obtain the dimensionless forms Eq. (1)-(4), we introduce

$$\begin{aligned}
x' &= x/x_0 && \text{with} && x_0 = \sqrt{D_\mathrm{g}/k_\mathrm{d}}\\
t' &= t/t_0 && \text{with} && t_0 = 1/k_\mathrm{d}\\
G &= c_\mathrm{g}/g_0 && \text{with} && g_0 = b_0\\
B &= c_\mathrm{b}/b_0 && \text{with} && b_0 = \sqrt{k_\mathrm{d}k_\mathrm{i}/k_\mathrm{p}}\\
F &= c_\mathrm{f}/f_0 && \text{with} && f_0 = b_0\\
I &= c_\mathrm{i}/i_0 && \text{with} && i_0 = k_\mathrm{i}
\end{aligned}$$

and respectively,

$$\begin{aligned} \tilde{D}_\mathrm{b} &= D_\mathrm{b}/D_\mathrm{g} & & \\ \tilde{k}_\mathrm{i1} &= k_\mathrm{i1}/(k_\mathrm{d}k_\mathrm{i}) & \text{and} \quad & \tilde{k}_\mathrm{f1} = \tfrac{k_\mathrm{f1}}{k_\mathrm{d}k_\mathrm{i}} \\ \tilde{k}_\mathrm{i2} &= k_\mathrm{i2}/k_\mathrm{d} & \text{and} \quad & \tilde{k}_\mathrm{f2} = k_\mathrm{f2}/k_\mathrm{d}. \\ \tilde{D}_\mathrm{i} &= D_\mathrm{i}/D_\mathrm{g} & & \end{aligned}$$

In the main text we dropped the tildes.

Supplementary Note 3

Fixed points. At the spatially homogeneous fixed points of the system Eq. (1)-(4) the CDR incorporated actin B takes the following values

$$B_0^* = 0 \tag{2a}$$

$$B_{1\pm}^* = \frac{(A-a)}{2} \pm \sqrt{\frac{(A-a)^2}{4} - (1+\alpha)}. \tag{2b}$$

Here $A = G^* + B^* + F^*$ is the total actin density in the system, $a = \tilde{k}_\mathrm{i1}/\tilde{k}_\mathrm{i2}$ the ratio of the kinetic constants of the inhibitory complex I, and $\alpha = \tilde{k}_\mathrm{f1}/\tilde{k}_\mathrm{f2}$ the ratio of the kinetic constants of the cortical and stress fiber actin F. The fixed point with B_0^* is always stable, whereas $B_{1\pm}^*$ undergoes the bifurcations that are described in the main text. The respective other components of the fixed point are:

$$F_{0,1\pm}^* = \alpha \frac{A - B_{0,1\pm}^*}{\alpha + (1 + aB_{0,1\pm}^*)} \tag{3a}$$

$$G_{0,1\pm}^* = A - B_{0,1\pm}^* - F_{0,1\pm}^* \tag{3b}$$

$$I_{0,1\pm}^* = aB_{0,1\pm}^*. \tag{3c}$$

Supplementary Note 4

Bistability of counter propagating front solutions. To demonstrate the counter-propagation of wavefronts, we initiate half of the domain (-60, 0) at the stable fixed point $\mathbf{P}_0^*$ and the other half (0, 60) at the fixed point $\mathbf{P}_{1+}^*$. Supplementary Fig. 2A shows a close up of the profile around the origin of the domain. With this initial condition, the system evolves as $\mathbf{P}_{1+}^*$ invades the

state $\mathbf{P}_0^*$ (Supplementary Fig. 2B). This situation corresponds to the expansion phase of CDRs.

Next, we make a small, localized perturbation in the field G (in form of a Gauss curve of negative amplitude, Supplementary Fig. 2D), all other fields are unchanged. From this initial condition the front propagates into the reverse direction (Supplementary Fig. 2E), with $\mathbf{P}_0^*$ invading $\mathbf{P}_{1+}^*$.

In the bistable regime the robust mechanism of wave reversal at boundaries persists and waves will continue to collapse back to points, regardless of the domain geometry. Supplementary Fig. 3 shows a time-lapse of wave dynamics on an asymmetric domain.

Experimentally, CDRs colliding head on are observed to mutually annihilate locally forming fused CDRs [2]. The bistability in our model naturally gives rise to this dynamics upon wavefront collisions as shown in Supplementary Fig. 4.

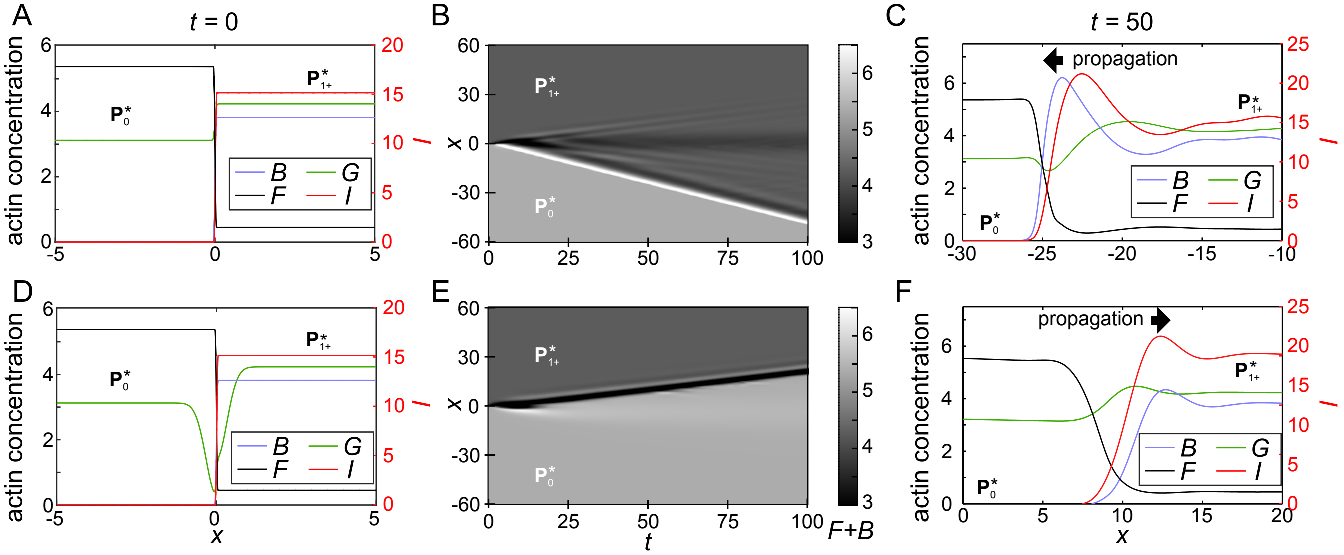

Supplementary Figure 2: *Counter-propagating front solutions.* (A-C) Front solutions in which the state $\mathbf{P}_{1+}^*$ invades $\mathbf{P}_0^*$. (A) Initial profiles of the fields around the origin of the domain (-60, 60). (B) kymograph. (C) profiles of the fields at $t = 50$. (D-F) The respective plots for a front solution in which the state $\mathbf{P}_0^*$ invades $\mathbf{P}_{1+}^*$. Note that the only difference between the two simulations is the slight deviation of the G field in the initial profile (compare (A) and (D)). Parameters: $D_b = 0.12$, $k_{i1} = 2.09$, $k_{i2} = 0.53$, $k_{f1} = 2.05$, $k_{f2} = 1.19$, $A = 8.5$, domain length: 60, grid size: 0.05.

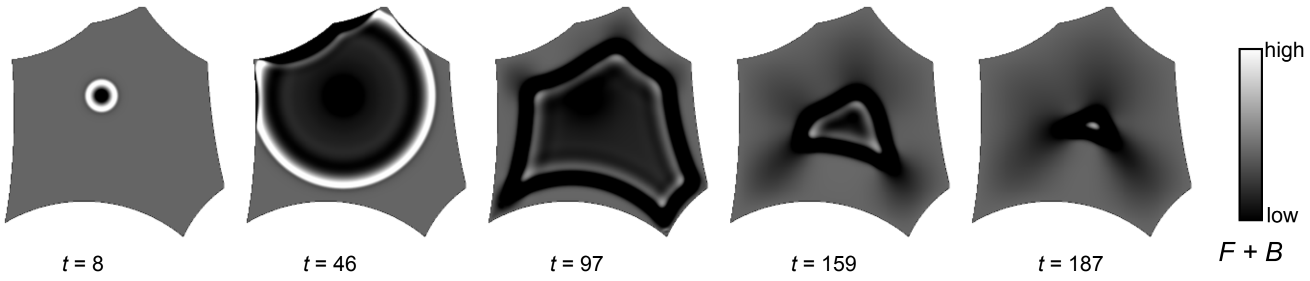

Supplementary Figure 3: *Dynamics of the system in the bistable regime on an asymmetric domain.* Time stamps are with respect to the start of the simulation at $t = 0$. Parameters: $D_{\mathrm{b}} = 0.12$, $k_{\mathrm{i1}} = 2.09$, $k_{\mathrm{i2}} = 0.53$, $k_{\mathrm{f1}} = 2.05$, $k_{\mathrm{f2}} = 1.19$, $A = 9.67$, maximal domain diameter: ca. 50, maximal mesh size: 0.5.

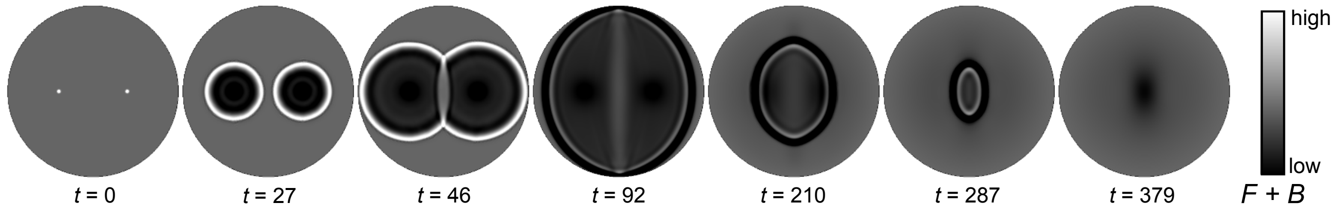

Supplementary Figure 4: Numerical results exhibiting wavefront collision of initially two expanding ring-shaped wavefronts. Upon collision the wavefronts mutually locally annihilate giving rise to one fused structure. Parameters: $D_{\mathrm{b}} = 0.12$, $k_{\mathrm{i1}} = 2.09$, $k_{\mathrm{i2}} = 0.53$, $k_{\mathrm{f1}} = 2.05$, $k_{\mathrm{f2}} = 1.19$, $A = 9.67$, domain radius: 50, maximal mesh size: 0.5.

Supplementary Note 5

Simulation of growth factor stimulated CDRs. It is a common strategy in the research on CDRs to stimulate their formation via growth factors such as PDGF [3, 4]. Cells utilize receptor tyrosine kinsases to transmit specific binding events between growth factors and the respective receptors into the cell interior where signaling cascades are triggered that lead to CDR formation. Since growth factors are not spatially confined to specific regions of the cell membrane stimulation results in a spatially extended region of excitation. Correspondingly, upon growth factor stimulation CDRs are usually not observed to initiate from points but spatially extended excited regions (see, e.g., Supplementary Fig. 5*A*).

We simulate the respective behavior by excitation of system (Eq. (1)-(4)) by a field of normally distributed random values. Similar to the behavior of CDRs upon growth factor stimulation, the system initially forms small short-living wave structures and eventually one ring-shaped front surrounding the excited domain (Supplementary Fig. 5*B*). The front contracts and collapses to one point, which is in accord with the dynamics of CDRs after growth factor simulation.

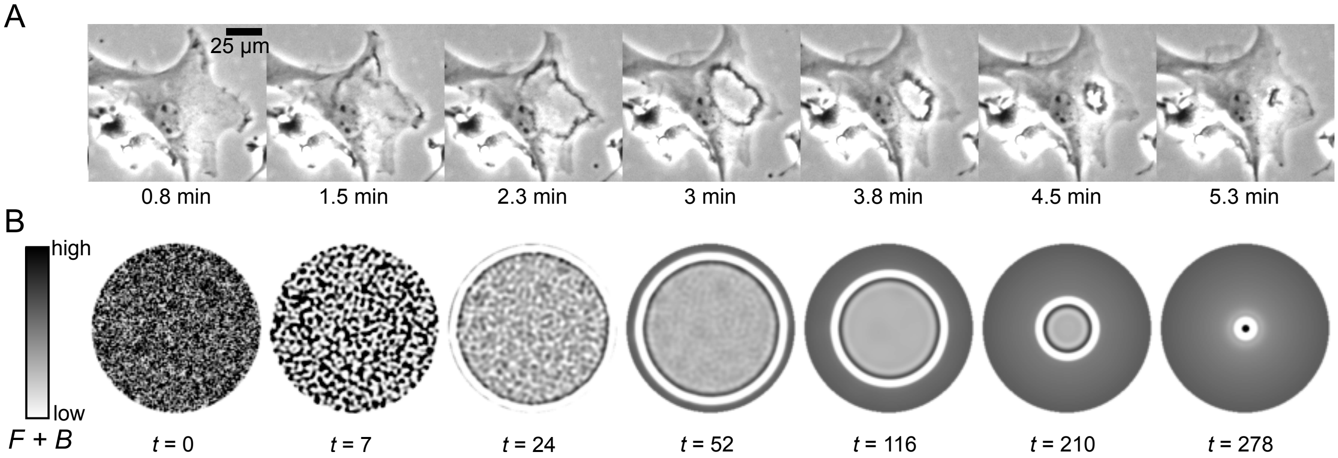

Supplementary Figure 5: *Simulating growth factor-induced CDR formation.* (*A*) Growth factor-induced CDR formation. Time points with respect to growth factor addition (PDGF). (*B*) Dynamics of the system upon stimulation with a field of random numbers. Parameters: $D_b = 0.12$, $k_{i1} = 2.09$, $k_{i2} = 0.53$, $k_{f1} = 2.05$, $k_{f2} = 1.19$, $A = 9.67$, domain radius: 50, maximal mesh size: 0.5.

Supplementary References

[1] J. Hasegawa, K. Tsujita, T. Takenawa, and T. Itoh, "Arap1 regulates the ring size of circular dorsal ruffles through arf1 and arf5," *Mol. Biol. Cell*,

vol. 23, no. 13, pp. 2481–89, 2012.

[2] E. Bernitt, C. G. Koh, N. Gov, and H.-G. Döbereiner, “Dynamics of actin waves on patterned substrates: A quantitative analysis of circular dorsal ruffles,” *PLoS ONE*, vol. 10, no. 1, p. e0115857, 2015.

[3] T. Itoh and J. Hasegawa, “Mechanistic insights into the regulation of circular dorsal ruffle formation,” *J. Biochem*, vol. 153, no. 1, pp. 21–29, 2012.

[4] J.-L. Hoon, W.-K. Wong, and C.-G. Koh, “Functions and regulation of circular dorsal ruffles,” *Mol. Cell. Biol.*, vol. 32, no. 21, pp. 4246–57, 2012.
